# RNA sequencing identifies *MAP1A* and *PTTG1* as predictive genes of aging CD264^+^ human mesenchymal stem cells at an early passage

**DOI:** 10.1007/s10616-025-00724-8

**Published:** 2025-02-19

**Authors:** Margaret K. Giler, H. Alan Tucker, Amanda K. Foote, Avery G. Francis, Sean D. Madsen, Yao-Zhong Liu, Kim C. O’Connor

**Affiliations:** 1 Department of Chemical and Biomolecular Engineering, School of Science and Engineering, Tulane University, New Orleans, LA USA; 2 Center for Stem Cell Research and Regenerative Medicine, School of Medicine, Tulane University, New Orleans, LA USA; 3 Department of Biostatistics and Data Science, School of Public Health and Tropical Medicine, Tulane University, New Orleans, LA USA; 4 Center for Aging, School of Medicine, Tulane University, New Orleans, LA USA

**Keywords:** CD264, *MAP1A*, *PTTG1*, Mesenchymal stem cells, Aging, RNA sequencing

## Abstract

Molecular profiles of mesenchymal stem cells (MSCs) are needed to standardize the composition and effectiveness of MSC therapeutics. This study employs RNA sequencing to identify genes to be used in concert with CD264 as a molecular profile of aging MSCs at a clinically relevant culture passage. CD264^−^ and CD264^+^ populations were isolated by fluorescence-activated cell sorting from passage 4 MSC cultures. CD264^+^ MSCs exhibited an aging phenotype relative to their CD264^−^ counterpart. Donor-matched CD264^−/+^ mRNA samples from 5 donors were subjected to pair-ended, next-generation sequencing. An independent set of 5 donor MSCs was used to validate differential expression of select genes with quantitative reverse transcription PCR. Pairwise differential expression analysis identified 2,322 downregulated genes and 2,695 upregulated genes in CD264^+^ MSCs relative to donor-matched CD264^−^ MSCs with a Benjamini–Hochberg adjusted *p*-value (BH *p*_*adj*_) < 0.1. Nearly 25% of these genes were unique to CD264^−/+^ MSCs and not differentially expressed at a significance level of BH *p*_*adj*_ < 0.1 in previous RNA sequencing studies of early- vs. late-passage MSCs. Least Absolute Shrinkage and Selection Operator regression identified microtubule-associated protein 1A (*MAP1A*) and pituitary tumor-transforming gene 1 (*PTTG1*) as predictive genes of CD264^+^ MSCs. Combined *MAP1A* and *PTTG1* expression correctly classified CD264 status of MSC samples with an accuracy of 100%. Differential expression and predictive ability of *MAP1A* and *PTTG1* compared favorably with that of existing senescence markers expressed in early passage CD264^−/+^ MSCs. This study provides the first linkage of *MAP1A* to CD264, aging and senescence. Our findings have application as quality metrics to standardize the composition of MSC therapies and as molecular targets to slow/reverse cellular aging.

**Supplementary Information:**

The online version contains supplementary material available at 10.1007/s10616-025-00724-8.

## Introduction

Numerous clinical trials have utilized mesenchymal stem cells (MSCs) to rejuvenate tissue and regulate the immune system (Rodríguez-Fuentes et al. 2021). Cellular heterogeneity of MSCs slows their translation into the clinic by contributing to variable trial results (Chaput et al. 2018; Mastrolia et al. 2019). Cell-to-cell variability in the therapeutic properties of MSCs arises in vivo, differs among donors and tissues, and is compounded by inconsistencies in biomanufacturing (Costa et al. 2021; Liu et al. 2017; Wilson et al 2019). There is a critical need for molecular profiles of MSC heterogeneity to regulate cell composition and manufacture MSC therapies with predictable treatment outcomes (O’Connor 2019).

We recently discovered that CD264 is a biomarker of aging cells in heterogenous cultures of human bone marrow MSCs. CD264 is a decoy receptor that inhibits cell death induced by tumor necrosis factor-related apoptosis-inducing ligand (Pan et al. 1998). CD264^+^ MSCs exhibit an aging phenotype and diminished stem cell fitness relative to CD264^−^ MSCs (Madsen et al. 2017, 2020). The content of CD264^+^ cells is variable in early-passage MSC cultures (Madsen et al. 2017). CD264 is a marker of an early stage of MSC aging, which is upregulated concurrently with p21 and remains elevated as aging progresses to senescence (Madsen et al. 2017). Culture-matched CD264^−^ and CD264^+^ MSCs have similar in vivo survival (Madsen et al. 2020).

This study employs RNA sequencing (RNAseq) to identify genes to be used in concert with CD264 as a predictive profile of aging MSCs at an early passage. Gene expression profiles reveal differences in cell populations that are undetectable with an immunophenotype alone. A gene profile can be a better predictor of cell function than immunophenotype (Gutierrez-Garcia et al. 2011). Furthermore, two distinct cell types can have nearly identical immunophenotypes (Vega et al. 2005). A secondary goal of this RNAseq study is to gain insight into underlying changes in pathway expression in CD264^−/+^ MSCs. The genes and pathways identified here have utility as potential quality metrics to standardize biomanufacturing of MSC therapies and molecular targets to slow/reverse cellular aging.

Previous RNAseq profiling of aging MSCs investigated differential gene expression between early- and late-passage cultures (Fernandez-Rebollo et al. 2020; Hänzelmann et al. 2015; Wang et al. 2021). One problem with this approach is that late passage is not clinically relevant. Typically, only early-passage MSCs are employed for therapeutic applications: two to five passages are the norm (Liu et al. 2017). Another problem is that the MSC cultures were heterogeneous. Variation in culture composition could have obscured some changes in gene expression.

The use of CD264 as an aging marker enables for the first time the study of differential gene expression between defined populations of aging and robust cells in the same culture of early-passage MSCs. With this approach, our research discovered numerous differentially expressed genes (DEGs) in CD264^−/+^ MSCs that were unreported in previous RNAseq studies of early- vs. late-passage MSCs. One of the predictor genes that we identified has no previous links to CD264, aging or senescence.

## Materials and methods

### MSC cultures

Primary MSCs were harvested from human bone marrow aspirate of healthy donors after approval by the institutional review boards at Tulane University, Texas A&M University and Baylor Scott & White Hospital. Passage 0 (P0) designates plastic adherent MSCs that have not been expanded. Unless otherwise stated, all supplies were ordered from Thermo Fisher Scientific (Waltham, MA, USA). MSCs were inoculated into T-flasks at a density of 100 cells/cm^2^ and maintained in a humidified incubator at 37 °C with 5% CO_2._ The cultures were expanded in complete culture medium with antibiotics (CCMA), which consisted of minimum essential medium alpha supplemented with 20% fetal bovine serum, 2 mM L-glutamine, 100 units/ml penicillin and 100 µg/ml streptomycin (Sekiya et al. 2002). Medium was completely exchanged every 3–4 days. MSCs were harvested from culture for passaging or analysis before they reached 50% confluency. Exposure to 0.25% trypsin/1 mM EDTA for 3 min was used for cell dissociation from the culture surface. Cell viability was evaluated with trypan blue exclusion and was ≥ 90%. MSCs were expanded to passage 4 for flow cytometric analysis and fluorescence-associated cell sorting (FACS) into CD264^−/+^ populations as described in Online Resource 1.

### RNA preparation and sequencing

RNA extraction was performed immediately after sorting (Tighe and Held 2010). P4 MSCs were washed with PBS and centrifuged at 2000 g for 12 min. The supernatant was discarded, and RNA was extracted with the RNeasy Plus Mini Kit (Qiagen, Germantown, MD, USA). The freshly extracted RNA was treated with RNase-Free DNase Set (Qiagen). Two technical replicates from each sorted culture were combined and then purified with RNAClean XP beads on the SPRIStand magnetic tube stand (Beckman Coulter, Brea, CA, USA). Total RNA was assessed for purity by quantifying the 260 nm/230 nm and 260 nm/280 nm ratios using the DS-11 Spectrophotometer/ Fluorometer (DeNovix, Wilmington, DE, USA). RNA integrity number was measured with the TapeStation 4150 (Agilent Technologies, Santa Clara, CA, USA) using an Agilent RNA ScreenTape.

Library preparation and sequencing were done by the Tulane NextGen Sequencing Core. The TruSeq Stranded mRNA Sample Preparation Kit (Illumina, San Diego, CA, USA) was used to purify poly-A mRNA from 0.3 µg total RNA per sample and then to convert fragmented mRNA into cDNA libraries containing TruSeq RNA CD indexes (Illumina). Final cDNA libraries were quantitated using the Qubit dsDNA HS assay kit. The size and concentration of the libraries were determined by running each library on the Agilent TapeStation 4150 using the Agilent D1000 ScreenTape. Smear analysis was performed using Agilent TapeStation Software v4.1.1 with a range of 200–600 base pairs to determine the average size of each library. All libraries were pooled at a final concentration of 750 pM with a spike-in of 1% PhiX control library v3 (Illumina) and loaded on an Illumina NextSeq P2 300 reagent cartridge. Paired-end and dual indexing sequencing (150 × 8 × 8 × 150 base pairs) was performed on the NextSeq 2000 (Illumina), yielding ~ 66 million paired-end reads per sample. Fastq files generated by Illumina BaseSpace DRAGEN Analysis Software v1.2.1 were used for further data analysis.

### Count matrix generation

Our RNAseq data was submitted to the National Center for Biotechnology Information Gene Expression Omnibus (accession number GSE247950), and RNAseq data from early- and late-passage MSCs was downloaded for comparative analysis (GSE59966, GSE125632, GSE178514). The FastQC program (https://www.bioinformatics.babraham.ac.uk/projects/fastqc/) was used to check the overall quality of the RNAseq raw data (fastq files). FastQC and downstream analysis were performed with default parameters, unless indicated otherwise. The Salmon program v1.9.0 (Patro et al. 2017) and the human reference transcriptome (Homo_sapiens.GRCh38.cdna.all.fa) were employed for transcript quantification analysis of the fastq files. A raw count matrix of gene expression was then generated with Bioconductor’s tximport package (Soneson et al. 2015).

### Differential expression analysis

The DESeq2 package v1.34.0 was used for pairwise analysis of the count matrix from donor-matched CD264^−^ and CD264^+^ samples (Love et al. 2014). The package utilizes the Wald statistic to identify differentially expressed genes between samples. The Generally Applicable Gene-set Enrichment (GAGE) method in the Bioconductor package was employed to discover pathways that were differentially regulated in CD264^−^ and CD264^+^ samples (Luo et al. 2009). GAGE accesses the Kyoto Encyclopedia of Genes and Genomes (KEGG) database as a pathway reference. Using the raw count matrix as input, GAGE performs a meta-test that summarizes the *t*-test statistics for differential expression of each gene in a pathway. Up- or down-regulated genes were submitted to the Database for Annotation, Visualization and Integrated Discovery (DAVID) to identify enriched annotation terms from the KEGG pathway, Gene Ontology (GO) and Universal Protein Resource (UniProt) databases (Dennis et al. 2003). The submitted genes had a Benjamini–Hochberg adjusted *p*-value (BH *p*_*adj*_) < 0.1, as determined by DESeq2 (Love et al. 2014). DAVID uses a modified version of the Fisher exact probability test to identify enriched terms (Hosack et al. 2003).

### Gene selection

The GLMNET package was employed to select genes for binary classification of MSC samples (Friedman et al. 2010). The package uses penalized maximum likelihood to fit generalized linear models and the Least Absolute Shrinkage and Selection Operator (LASSO) regression analysis for variable selection and regularization. The input to the program was the set of normalized and transformed differentially expressed genes with a |log_2_(fold change)|> 1 and a Bonferroni *p*_*adj*_ < 0.05. DESeq2 was used to normalize the counts based on both size factor and average transcript length. The normalized counts were then transformed so that CD264^−^ and CD264^+^ counts from the same donor were centered around 0 (Stanfill et al. 2019). The cv.glmnet function was run with a tenfold cross validation and repeated 100 times in R. After each run, the penalty parameter corresponding to the minimum misclassification error was selected, and the coefficient of each gene was determined. Genes that had non-zero coefficient values in more than 90% of the runs were selected for binary classification of MSC samples.

### Other methods

Supplementary Methods and Tables S1–S3 in Online Resource 1 provide other assays and statistical analysis.

## Results

### Characterization of CD264^−/+^ MSCs

**Fig. 1:**
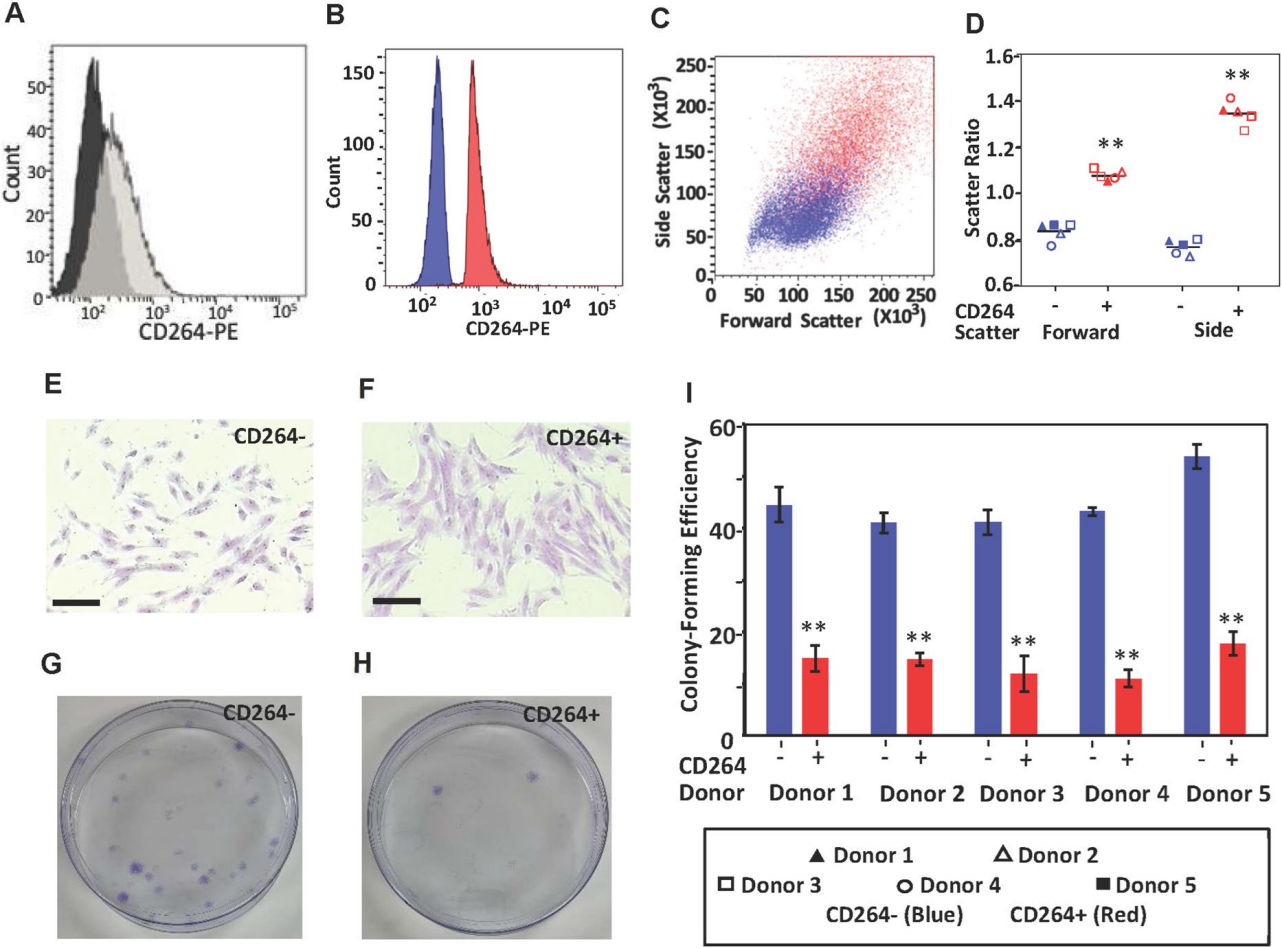
Quality assessment of FACS-sorted MSCs for RNA sequencing. **A**–**C** Representative histograms. **A**,** B** and scatter plot, **C** of passage P4 MSCs (18–20 cumulative doublings) labeled with anti-CD264-PE monoclonal antibody (*n* = 10,000 cells). **A** Isotype (black) and parent culture (light gray). **B-D** CD264^−^ and CD264^+ ^MSCs were immediately re-analyzed after sorting for CD264 expression (**B**) and scatter properties (**C**,** D**). **D** Scatter ratio of sorted MSCs relative to parent culture. Data are averaged over two technical replicates per CD264 group for each donor (SEM ≤ 7% of the mean, *n* = 5 donors). Mean values depicted as bars. **E**,** F** Representative images of sorted MSCs stained with crystal violet. **G**–**I** Efficiency of sorted MSCs to form colonies was evaluated in 10 cm cell culture dishes. **I **Colony-forming efficiency of CD264^−/+^ MSCs was determined for five donors. Data presented as the mean ± SEM of *n* = 5–6 dishes per donor, with each dish counted independently by two people. Scale bar = 200 μm. **p < 0.001 vs. CD264^−^ MSCs. *FACS* fluorescence-associated cell sorting, *MSC* mesenchymal stem cell, *PE* phycoerythrin

This study was performed with human bone marrow MSCs from 10 donors that were divided into a sequencing and validation set (Supplementary Fig. S1 in Online Resource 1). Our cells exhibited the immunophenotype, potency and plastic adherence that typify human MSCs (Supplementary Fig. S1 and Table S1 in Online Resource 1, Dominici et al. 2006). We investigated P4 MSCs (18–20 cumulative doublings) from 24- to 37-year-old donors since passage 4 is representative of MSCs in clinical trials (Liu et al. 2017) and since this age group produces MSCs containing a mixture of CD264^−^ and CD264^+^ cells. CD264^+^ cell content in our heterogeneous MSC cultures was 40% on average (Supplementary Fig. S1 in Online Resource 1), consistent with our previous findings (Madsen et al. 2017, 2020). This heterogeneity enabled pairwise comparisons of differential gene expression in donor-matched CD264^−^ and CD264^+^ cell populations generated by FACS. Sorted CD264^−^ and CD264^+^ MSC populations are defined here as having a CD264^+^ cell content of < 1% and > 95%, respectively, by flow cytometric analysis (Fig. 1A and B). FACS-sorted CD264^+^ MSCs exhibited an aging phenotype with an enlarged, granular morphology (Fig. 1C–F), less colony formation (Fig. 1E–I) and greater senescence-associated β-galactosidase (SA β-gal) activity (Supplementary Fig. S2 in Online Resource 1) than their CD264^−^ counterpart.

### Identification of differential gene and pathway expression in CD264^−/+^ MSCs

**Fig. 2:**
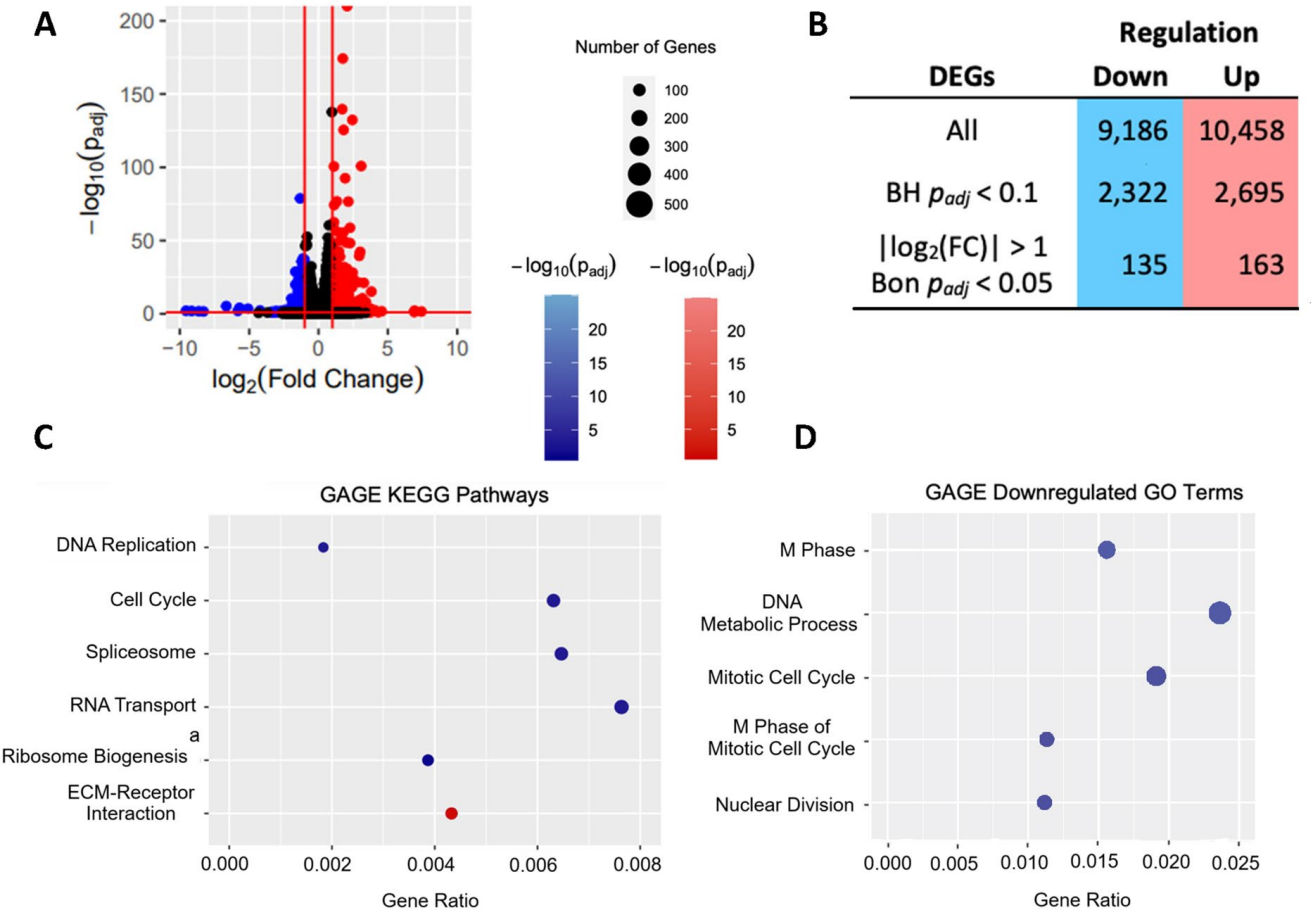
Differential expression analysis of CD264^−/+^ MSCs. Following paired-end mRNA sequencing of sorted CD264^−/+^ populations at passage 4, reads were mapped to the human genome and analyzed for differential expression between donor-matched pairs from *n* = 5 donors. Down- and upregulated genes, pathways and GO terms for CD264^+^ MSCs are presented relative to CD264^−^ control. **A** Volcano plot highlighting genes with |log_2_(fold change)|> 1 and BH *p*_adj_ < 0.1. **B** Table illustrating the effects of constraining *p*_*adj*_ and/or log_2_(fold change) on gene number. **C**,** D** Gene raw counts were analyzed by GAGE: dot plots of (**C**) KEGG pathways with BH *p*_adj_ < 0.1 and **D** the most significantly downregulated GO terms with BH *p*_adj_ < 0.1. All terms downregulated in CD264^+^ populations are in blue and upregulated in CD264^+^ populations are in red. ^a^Ribosome biogenesis in eukaryotes. *BH* Benjamini-Hochberg, *Bon* Bonferroni, *ECM* extracellular matrix, *FC* fold change, *GAGE* generally applicable gene-set enrichment, *GO* gene ontology, *KEGG* Kyoto Encyclopedia of Genes and Genomes, *MSC* mesenchymal stem cell. (Color figure online)

Sequencing mRNA from sorted P4 MSCs produced paired-end reads that mapped to the human genome with a 94–96% efficiency (Supplementary Table S4 in Online Resource 1). We followed a well-established RNAseq workflow for differential gene expression and downstream analysis of CD264^−/+^ MSCs (Love et al. 2019; Luo 2024; Rosati et al. 2024). The count matrix generated from the reads was analyzed with DESeq2 to detect DEGs in donor-matched CD264^−^ and CD264^+^ MSCs. DESeq2 is recognized for its high sensitivity and precision in predicting DEGs (Love et al. 2014). DESeq2 determined that 2,322 genes were downregulated and 2,695 genes were upregulated in CD264^+^ MSCs relative to their CD264^−^ counterpart (BH *p*_adj_ < 0.1, Fig. 2A and B, Online Resource 2). Of those genes, 135 downregulated genes and 163 upregulated genes in CD264^+^ MSCs satisfied the stringent threshold of a |log_2_(fold change)|> 1 and Bonferroni *p*_adj_ < 0.05 (Fig. 2B).

GAGE analysis identified how individual genes cooperated to differentially regulate pathways. This method was chosen for its ability to detect statistically and biologically relevant regulated pathways (Luo et al. 2009). GAGE identified six differentially regulated KEGG pathways in sorted P4 CD264^−/+^ MSCs (BH *p*_adj_ < 0.1, Fig. 2C). DNA replication and cell cycle were among the downregulated pathways in CD264^+^ MSCs, in agreement with the slower proliferation that we reported for this cell population (Madsen et al. 2017). The remaining downregulated pathways in CD264^+^ MSCs were associated with RNA processing at the level of ribosome biogenesis, splicing and transport. Downregulated RNA processing in CD264^+^ MSCs is consistent with the causal role of ribosome biogenesis in cell proliferation (Volarevic et al. 2000), dysregulation of splicing factor expression in senescent cells (Latorre et al. 2018), and impaired nuclear export of mRNA during cellular aging (Park et al. 2022). Extracellular matrix-receptor interaction was the only significantly upregulated KEGG pathway in CD264^+^ MSCs relative to donor-matched CD264^−^ MSCs. The composition of the extracellular matrix influences cell entrance into senescence, and conversely senescent cells cause changes to the matrix (Levi et al. 2020).

Functional annotation analysis supports the differentially expressed pathways in Fig. 2C. GAGE (Fig. 2D) and DAVID (Supplementary Fig. S3 in Online Resource 1) identified DNA replication and the cell cycle among the most significantly enriched terms in downregulated DEGs in CD264^+^ MSCs relative to CD264^−^ MSCs. DAVID identified several highly significant terms related to downregulated RNA processing in P4 CD264^+^ MSCs, such as mRNA splicing and the spliceosome (Supplementary Fig. S3 in Online Resource 1). Only DAVID analysis detected terms with BH *p*_*adj*_ < 0.1 for upregulated DEGs in CD264^+^ MSCs. The most significant of which include cell adhesion, collagen binding and the extracellular matrix (Supplementary Fig. S3 in Online Resource 1), which support the upregulated GAGE pathway for extracellular matrix-receptor interactions in CD264^+^ MSCs (Fig. 2C).

### Comparison of RNAseq data from CD264^−/+^ MSCs and early/late-passage MSCs

DEGs in P4 CD264^+^ MSCs vs. donor-matched CD264^−^ MSCs at the same passage were compared to DEGs in late-passage cultures of heterogeneous MSCs vs. early-passage MSCs reported in previous bulk RNAseq studies. Raw RNAseq data was downloaded from the Gene Expression Omnibus repository for P2-4 vs. P11-13 MSCs from Fernandez-Rebollo et al. (2020, GSE125632), Hänzelmann et al. (2015, GSE59966) and Wang et al. (2021, GSE178514). The datasets were subject to the same processing and analysis used for CD264^−/+^ MSCs to avoid artifacts from different algorithms.

**Fig. 3:**
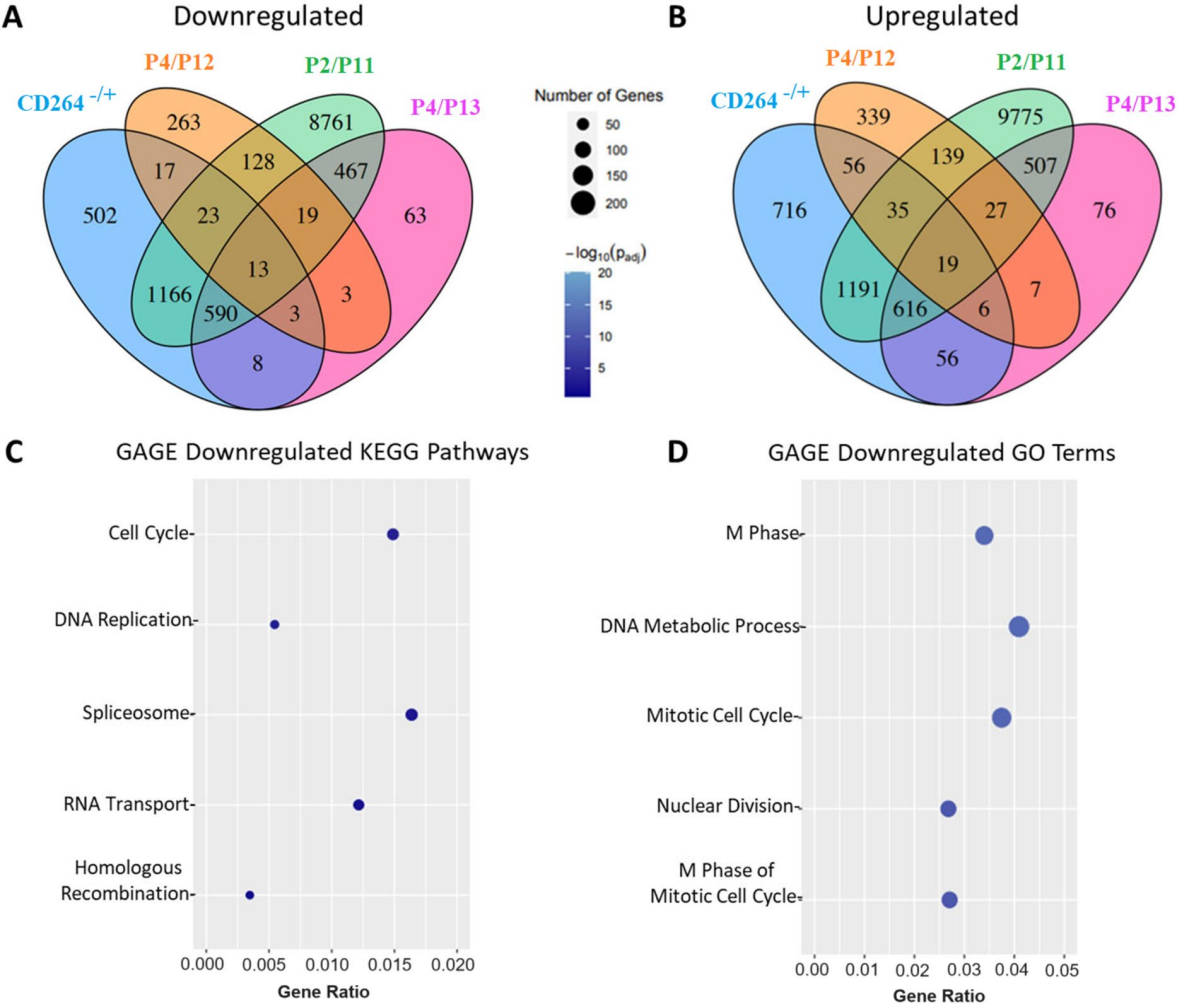
Comparison of differential gene expression in CD264^−/+^ MSCs with that from previous bulk RNAseq studies of early- vs. late-passage MSCs. Donor-matched cultures of CD264^−^ and CD264^+^ MSCs were at passage P4 (blue). Raw RNAseq data were downloaded from the National Center for Biotechnology Information Gene Expression Omnibus repository for P2-4 vs. P11-13 MSCs from Fernandez-Rebollo et al. (2020, GSE125632, green), Hänzelmann et al. (2015, GSE59966, pink) and Wang et al. (2021, GSE178514, orange). All datasets were generated from human bone marrow MSCs and were subject to the same mapping and differential expression analysis. Venn diagrams of **A** downregulated and **B** upregulated genes with BH *p*_*adj*_ < 0.1 in CD264^+^ MSCs and late-passage MSCs relative to their respective controls. **C**,** D** GAGE analysis of all differentially expressed genes that are fully shared in all four datasets. Dot plots of the most significantly downregulated KEGG pathways (**C**) and GO terms (**D**) for both CD264^+^ and late-passage MSCs. No KEGG pathways or GO terms were significantly upregulated for the fully shared genes. See Fig. 2 caption for nomenclature. (Color figure online)

Thirteen downregulated and 19 upregulated DEGs in P4 CD264^+^ MSCs were fully shared with late-passage MSCs in the three previous datasets, and 502 downregulated and 716 upregulated DEGs were unique to CD264^−/+^ MSCs (BH *p*_*adj*_ < 0.1, Fig. 3A and B, Online Resource 3). Most of the downregulated pathways and GO terms in Fig. 3 for the fully shared DEGs are the same as in Fig. 2 for all DEGs in CD264^−/+^ MSCs. These findings indicate that impaired DNA replication, cell cycle and RNA processing are common features of aging CD264^+^ MSCs at passage 4 and senescing MSCs at passage 11–13. GAGE analysis did not detect any significantly upregulated pathways or GO terms for the fully shared DEGs, and DAVID analysis did not detect any significantly enriched terms for these DEGs.

For DEGs unique to P4 CD264^−/+^ MSCs, DAVID analysis indicated that ubiquitination was prominent among the most significant biological process GO terms for downregulated DEGs in CD264^+^ MSCs relative to CD264^−^ controls (Supplementary Fig. S4 in Online Resource 1). Dysfunction of the ubiquitin–proteasome system is a major driver of cellular aging (Kevei and Hoppe 2014) and may have contributed to the accumulation of aging CD264^+^ cells in our P4 MSC cultures. Extracellular matrix was the subject of several of the most significant DAVID terms for upregulated DEGs in CD264^+^ MSCs (Supplementary Fig. S4 in Online Resource 1). Aging may have altered interactions of CD264^+^ MSCs with their microenvironment through the extracellular matrix. GAGE analysis was unable to identify any significantly regulated pathways or GO terms for DEGs unique to CD264^−/+^ MSCs.

### Selection and validation of predictive genes for CD264^−/+^ MSCs

Predictive genes were previously selected for other cell systems by applying an arbitrary cut-off to a statistical ranking (Yin et al. 2014). This approach does not account for multicollinearity among genes whose expression are highly correlated. To overcome this problem, we selected predictive genes with LASSO regression, which controls multicollinearity by shrinking coefficients for features with minor effects to zero (Tibshirani 1996). LASSO has accurately identified predictive genes and other features for a variety of cell systems (Cao et al. 2022; Liu et al. 2022).

**Fig. 4:**
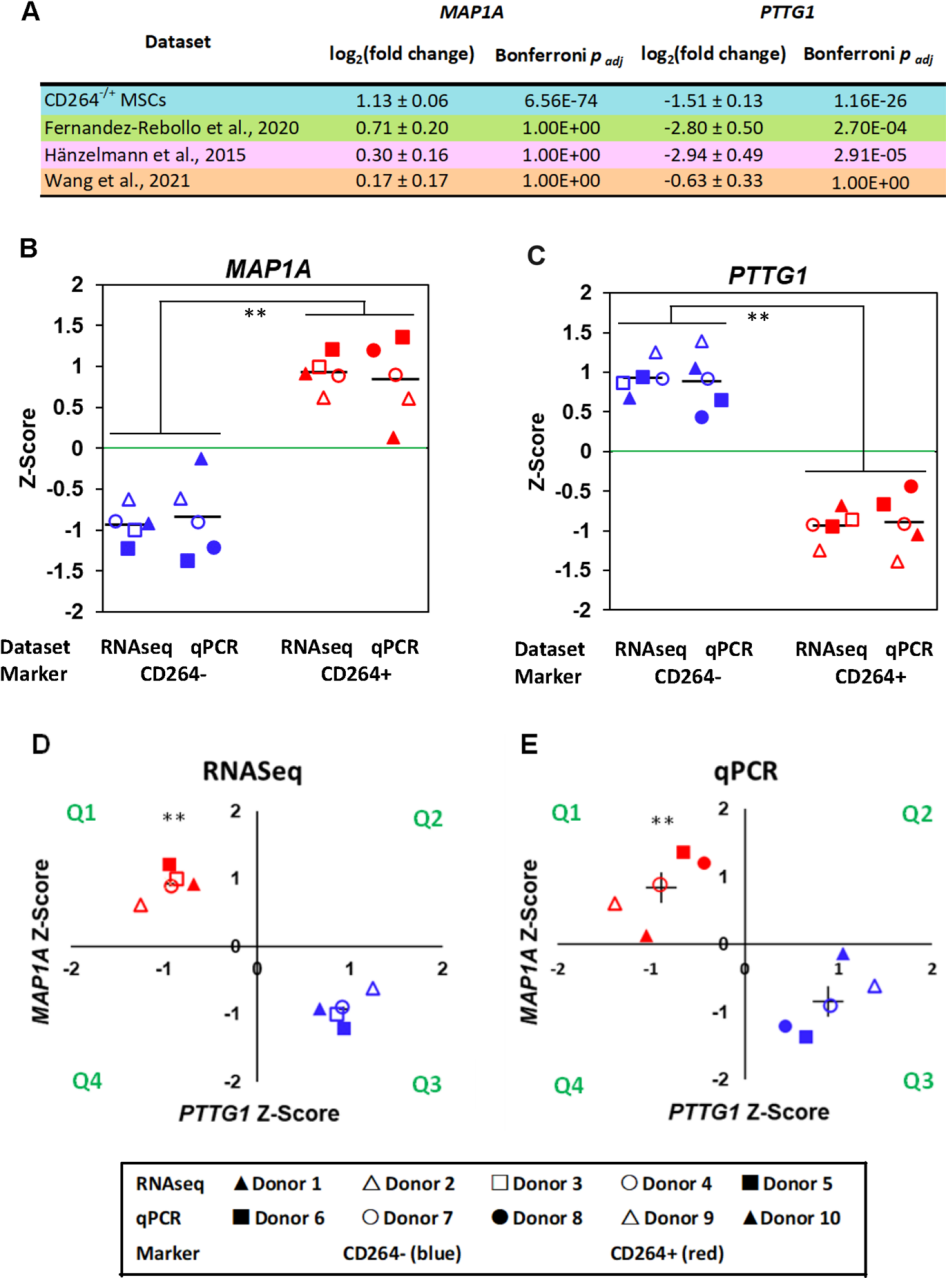
Validation of differential expression of LASSO-selected genes in CD264^−/+^ MSCs. **A** Comparison of differential expression of *MAP1A* and *PTTG1* among all four datasets in Fig. 3. DESeq2 estimates of mean log_2_(fold change) in gene expression with standard error for CD264^+^ vs. CD264^−^ MSCs and late- vs. early-passage MSCs. **B**, **C** Differential expression of (**B**) *MAP1A* and (**C**) *PTTG1* in FACS-sorted CD264^−^ and CD264^+^ populations assessed by RNAseq and qPCR on independent sets of donor MSCs at passage 4. Donor-matched RNAseq raw counts and qPCR cycle threshold numbers were normalized to the same scale with centered Z-scores. Mean values depicted as bars. **D**, **E** Separation of MSCs into distinct CD264^+^ and CD264^−^ groups in quadrants Q1 and Q3, respectively, based on the combined Z-scores of *MAP1A* and *PTTG1* for (**D**) RNAseq and (**E**) qPCR datasets. SEM < 5% of the mean for RNAseq dataset and depicted as bars centered on the mean for qPCR dataset. *PUM1*: housekeeping gene for qPCR. Sample size: *n* = 5 donors/dataset. **p ≤ 0.01 vs. CD264^−^ MSCs. Nomenclature: FACS, fluorescence-activated cell sorting; LASSO, Least Absolute Shrinkage and Selection Operator; *MAP1A*, microtubule-associated protein 1A; MSC, mesenchymal stem cell; *PTTG1,* pituitary tumor-transforming gene 1; Q, quadrant; qPCR, quantitative reverse transcription polymerase chain reaction; RNAseq, RNA sequencing

LASSO regression was performed on genes with a |log_2_(fold change)|> 1 and a Bonferroni *p*_*adj*_ < 0.05 as estimated by DESeq2. This log_2_(fold change) was chosen because it is a standard threshold of significance for gene expression that is verifiable via quantitative reverse transcription polymerase chain reaction (qPCR). The Bonferroni *p*_*adj*_ was used because it is more stringent than the BH *p*_*adj*_, and thus, limits analysis to genes with the most significant change in expression. Two datasets were used for LASSO input: all DEGs in P4 CD264^−/+^ MSCs (298 genes, Fig. 2B) and those from the significant KEGG pathways (32 genes, Fig. 2C). For each set, 100 iterations of LASSO were run to classify RNAseq samples as originating from CD264^−^ or CD264^+^ MSCs. The selected genes minimized the misclassification error in all 100 iterations. LASSO regression selected microtubule-associated protein 1A (*MAP1A*) from all DEGs and pituitary tumor transforming gene 1 (*PTTG1*) from significant pathway DEGs. DESeq2 estimated a log_2_(fold change) of 1.1 ± 0.1 and Bonferroni *p*_*adj*_ of 6.6E-74 for *MAP1A* in P4 CD264^+^ MSCs relative to their CD264^−^ counterpart (Fig. 4A). The corresponding values for *PTTG1* were -1.5 ± 0.1 and 1.2E-26, respectively. The same two genes were selected when a BH *p*_*adj*_ < 0.1 was used instead of a Bonferroni *p*_*adj*_ < 0.05 as an input threshold.

Our comparative analysis showed that *PTTG1* is among the shared DEGs with |log_2_(fold change)|> 1 and a Bonferroni *p*_*adj*_ < 0.05 for two of the three previous studies of early- vs. late-passage MSCs (Fig. 4A). *PTTG1* was consistently downregulated in CD264^+^ and late-passage MSCs. *MAP1A* expression was higher for late-passage MSCs in all three previous studies, but the change in expression was insignificant (Fig. 4A). In our study alone, differential *MAP1A* expression had an average |log_2_(fold change)|> 1 and Bonferroni *p*_*adj*_ < 0.05.

Predictive capacity of the LASSO-selected genes was validated with qPCR on sorted P4 CD264^−/+^ MSCs from an independent set of 5 donors. RNAseq raw counts and qPCR threshold cycle numbers were centered for pairwise comparisons between sorted groups and normalized to the same scale with Z-scores to facilitate comparison between RNAseq and qPCR datasets. Relative expression of each target gene was normalized against Pumilio RNA-binding family member 1 (*PUM1*), a reference gene for aging studies (González-Bermudez et al. 2019).

RNAseq and qPCR datasets had similar Z-scores for both *MAP1A* and *PTTG1* expression. For example, the *MAP1A* |Z-scores| for CD264^+^ vs. CD264^−^ MSCs were 0.93 ± 0.04 for RNAseq and 0.84 ± 0.22 for qPCR (Fig. 4B and C). CD264 expression had a significant effect on Z-scores for both LASSO-selected genes (*p* < 0.0001). There were no sex-linked differences in *MAP1A* or *PTTG1* expression. The combination of *MAP1A* and *PTTG1* Z-scores clustered MSC samples into distinct groups that had the correct CD264 classification with an accuracy of 100% (Fig. 4D and E). CD264^+^ clusters were significantly separated from CD264^−^ clusters due to a difference in the location of cluster centroids (*p* ≤ 0.01) and not a difference in cluster dispersion.

### Comparison of differential expression and predictive capacity of *MAP1A*, *PTTG1* and senescence markers

For this comparison, marker genes were selected that had been previously investigated in senescent MSCs (Bertolo et al. 2019, 2020; González-Gualda et al. 2021) and that were associated with differentially expressed pathways in CD264^−/+^ MSCs (Fig. 2C). We identified 10 existing senescence markers that satisfied these criteria (Table 1), and they are all involved in the downregulated cell cycle pathway in CD264^+^ MSCs (Fig. 2C). Three of the marker genes (*ANKRD1*, *CDKN1A* and *CDKN2A*) in Table 1 were upregulated at passage 4 in aging CD264^+^ MSCs relative to CD264^−^ MSCs; four were downregulated (*CDCA7*, *CDK1*, *CDKN2C* and *E2F1)*; and three had insignificant differential expression or were not detected by RNA sequencing in this study (*CCND2*, *RB1* and TP53).

**Table 1:** Comparison of differential gene expression and predictive values for *MAP1A*, *PTTG1* and senescence markers in CD264^−/+^ MSCs at passage 4

Gene	Log_2_ (fold change)^a^	Bonferroni *p*_adj_^a^	Predictive values^b^ (%)	
			Positive^c^	Negative^d^
Upregulated^e^				
*MAP1A*	1.13 ± 0.06	6.56E−74	100	100
*ANKRD1*	1.11 ± 0.08	1.99E−36	100	100
*CDKN1A*	1.81 ± 0.07	1.66E−125	100	100
*CDKN2A*	0.84 ± 0.11	7.36E−11	80	80
Downregulated^e^				
*PTTG1*	− 1.51 ± 0.13	1.16E−26	100	100
*CDCA7*	− 1.47 ± 0.14	1.81E−21	100	100
*CDK1*^*f*^	− 0.69 ± 0.14	2.08E−02	80	80
*CDKN2C*	− 0.91 ± 0.11	1.97E−12	100	100
*E2F1*	− 1.04 ± 0.12	6.19E−13	100	100
Insignificant fold change or not detected				
*CCND2*	Not detected			
*RB1*	− 0.16 ± 0.09	1.00E+00	60	60
*TP53*	− 0.05 ± 0.08	1.00E+00	60	60

^a^Fold change and adjusted *p*-value estimated with the DESeq2 package v1.34.0

^b^Predictive values for *n* = 10 samples from confusion matrices in Figs. S5 and S6

^c^Positive predictive value = true positives/(true positives + false positives)

^d^Negative predictive value = true negatives/(true negatives + false negatives)

^e^Expression in CD264^+^ MSCs relative to CD264^−^ MSCs

^f^Also known as CDC2

Table 1 shows fold change in expression and its significance, which were estimated with DESeq2 (*n* = 10 RNA sequencing samples from 5 donors). Predictive values in Table 1 were evaluated with confusion matrices constructed from the DESeq2 normalized counts of gene expression (Figs. S5  and S6). The confusion matrix mapped the predicted CD264 classification based on a ranking of the normalized counts to the actual CD264 classification of the MSC samples determined by flow cytometry. The positive predictive value is the percentage of positive predictions that are actual positives; the negative predictive value, the percentage of negative predictions that are actual negatives.

*MAP1A* and *PTTG1* had comparable or better differential expression and predictive capacity than many of the existing senescence markers in CD264^−/+^ MSCs at passage 4. For the upregulated genes in Table 1, only *CDKN1A* had a larger log_2_ (fold change) in expression (1.81 ± 0.07) than *MAP1A* (1.13 ± 0.06) and a smaller Bonferroni adjusted *p*-value (1.66E-125 for *CDKN1A* vs. 6.56E−74 for *MAP1A*). Both *CNKN1A* and *MAP1A* had positive and negative predictive values of 100% (*n* = 10 samples). These findings are supported by our earlier work which demonstrated a strong correlation between the expression of CD264 and p21, which is encoded by *CDKN1A* (Madsen et al. 2017). Relative to the downregulated senescence markers in Table 1, *PTTG1* had comparable or higher log_2_ (fold change) in expression (− 1.51 ± 0.13), the smallest Bonferroni *p*_*adj*_ (1.16E−26), and perfect predictive values for the 10 samples examined. Notably, six of the ten senescence markers in Table 1 failed to satisfy the thresholds of differential expression (|log_2_(fold change)|> 1) and/or significance (Bonferroni *p*_*adj*_ < 0.05) that we used in our LASSO analysis to select *MAP1A* and *PTTG1* as predictive genes of early passage CD264^−/+^ MSCs.

## Discussion

With RNAseq and LASSO analysis, we identified *MAP1A* and *PTTG1* as predictive genes to distinguish between CD264^*−*^ and aging CD264^+^ cells in P4 MSC cultures. Neither gene was discussed in published papers from the previous RNAseq studies of early- vs. late-passage MSCs (Fernandez-Rebollo et al. 2020; Hänzelmann et al. 2015; Wang et al. 2021). Interrogation of the data from these studies revealed similar trends in expression for both genes during passaging as reported here. Only in our study, however, did changes in *MAP1A* expression exceed accepted norms for thresholds in fold change and significance. MSC heterogeneity in the previous studies may have concealed differential *MAP1A* expression. Our detection of *MAP1A* as a predictive gene of CD264^+^ MSCs demonstrates the utility of our experimental design to discover novel aging genes by using well-defined cell populations.

### Predictive genes of MSCs at an early stage of aging

MSC aging is a continuous and organized process that culminates in senescence (Wagner et al. 2008). Our study identified *MAP1A* and *PTTG1* as predictive genes of a population of aging MSCs that have not become fully senescent. We previously showed that CD264 is a marker of an early stage of MSC aging: CD264^+^ MSCs undergo cell division, albeit slowly, and CD264 is upregulated before p16 during serial passage of MSCs (Madsen et al. 2017). Possibly, aging MSCs may be more effectively rejuvenated before they senesce than afterwards since there is less cellular dysfunction. If so, *MAP1A* and *PTTG1* would be useful to identify an MSC population amenable to rejuvenation and as potential targets to restore lost cellular function.

### Comparisons with existing senescence markers

Differential expression and predictive ability of *MAP1A* and *PTTG1* compared favorably with that of existing senescence markers expressed in early passage CD264^−/+^ MSCs. The fold change in expression, its significance and predictive value of *PTTG1* equaled or surpassed those of all senescence markers examined which were downregulated in CD264^+^ MSCs relative to CD264^−^ MSCs. *CDKN1A* was the only upregulated senescence marker whose differential expression exceeded that of *MAP1A* in both fold change and significance. More than half of the senescence markers investigated had expression that was below the thresholds in this study. These findings raise concern about employing established senescence markers to characterize cells at an early passage, such as CD264^+^ MSCs, that are aging but not yet fully senescent. Their expression, or lack thereof, may have limited usefulness. Instead, a new set of gene markers is warranted for cells at an early stage of aging.

### MAP1A

This study is the first direct link of *MAP1A* to CD264, cellular aging and senescence for any cell type. *MAP1A* is frequently investigated in the context of neurodegenerative diseases, including Alzheimer’s and Parkinson’s (Cai et al. 2023; Jiao et al. 2020). Insight into the differential expression of *MAP1A* in CD264^−/+^ MSCs comes from its function to promote microtubule polymerization in the cytoskeleton (Pazzagli & Avila 1994). Microtubule stabilization induces senescence in multiple cell types (Chu et al. 2022; Klein et al. 2005). Dynamic changes to the microtubule network are essential to chromosome movement during cell division (Vicente and Worderman 2019). Also, microtubules influence the cellular stress response by sequestering stress response proteins and transmitting stress signals through cytoskeletal remodeling (Parker et al. 2014). Perhaps *MAP1A* upregulation in CD264^+^ MSCs stabilizes microtubules to impede cell division and/or induces a stress response and cell survival during cellular aging. The latter is consistent with the potential role of CD264 in the survival of aging MSCs (Madsen et al. 2020).

### PTTG1

The literature is silent on the relationship of *PTTG1* to CD264. *PTTG1* is often examined for its role in cancer (Ren and Jin 2017). *PTTG1* is a member of the KEGG cell cycle pathway. It promotes accurate chromosome segregation during cell division by preventing premature separation of sister chromatids (Zou et al. 1999). There is conflicting data in the literature about *PTTG1* expression in MSCs and fibroblasts. We observed that *PTTG1* is downregulated in aging CD264^+^ MSCs. In support of our results, MSCs from *PTTG1*-null mice exhibited several key indicators of aging (Rubinek et al. 2007). Similar trends were observed by Menicanin et al. (2010) and Lee et al. (2014). Contrary to our findings, Hsu et al. (2010) found that *PTTG1* overexpression induced a senescent phenotype in normal human fibroblasts. A possible explanation for this apparent contradiction could be that deviation in *PTTG1* expression from the norm by either down- or upregulation may lead to genetic instability and cellular aging from erroneous chromosome segregation.

### Applications

Our findings have application as a quality attribute and as molecular targets during the manufacturing of MSC therapies. A combination of gene and surface marker expression can form a more robust molecular profile of a cell population than an immunophenotype alone (Gutierrez-Garcia et al. 2011; Vega et al. 2005). We envision that *MAP1A* and *PTTG1* expression will be used in concert with CD264 surface expression to identify aging cells in early-passage MSC cultures. This molecular profile has potential as a quality attribute to assess and control cell composition at all stages of MSC manufacturing from cell source selection, development of culture conditions, removal of aging cells during culture expansion, and evaluation of the final clinical product. Another application of our work is to identify molecular targets to slow and/or reverse cellular aging in MSCs. Differentially expressed genes and pathways in CD264^−/+^ MSCs can be targeted with pharmaceuticals or precision gene editing. For example, a small-molecule activator of the cell cycle pathway (Ito et al. 2019) could be a candidate rejuvenation drug for aging CD264^+^ MSCs to counteract the downregulation of *PTTG1* and other cell cycle genes. The net effect of quality assessment and molecular targeting is the potential for unprecedented control over the composition and effectiveness of MSCs therapies to improve patient outcomes.

### Future directions

Before the initial discoveries presented here can be translated to MSC manufacturing, the predictive capacity of *MAP1A* and *PTTG1* to identify aging MSCs at an early passage needs to be tested with a larger cohort of donors and in independent labs to assess the generalizability and reproducibility of our findings. Moreover, future research is required to ascertain the role of differential *MAP1A* and *PTTG1* expression in causing MSC aging. Knockdown and overexpression studies would determine the direct effect of these genes on the aging process. If the relationship were causal, *MAP1A* and *PTTG1* would be prime targets to slow/reverse MSC aging.

## Supplementary Information

Below is the link to the electronic supplementary material.

Supplementary file1 (PDF 2221 KB)

Supplementary file2 (XLSX 563 KB)

Supplementary file3 (XLSX 2510 KB)

## Data Availability

Datasets presented in this study can be found in the National Center for Biotechnology Information Gene Expression Omnibus (https://www.ncbi.nlm.nih.gov/geo/online repositories) under accession numbers GSE247950, GSE59966, GSE125632 and GSE178514.
