# Supplementary material for: RNA sequencing identifies MAP1A and PTTG1 as predictive genes of aging CD264+ human mesenchymal stem cells at an early passage
Source: Cytotechnology. 2025 Feb 19;77(2):63. doi: 10.1007/s10616-025-00724-8 (PMC11839963; doi:10.1007/s10616-025-00724-8)
Supplement: Supplementary file 1 — Supplementary file1 (PDF 2221 KB) [file 10616_2025_724_MOESM1_ESM.pdf]

RNA sequencing identifies *MAP1A* and *PTTG1* as predictive genes of aging CD264$^+$ human mesenchymal stem cells at an early passage

Margaret K. Giler • H. Alan Tucker • Amanda K. Foote • Avery G. Francis • Sean D. Madsen • Yao-Zhong Liu • Kim C. O'Connor

Corresponding Author:
Kim C. O'Connor
Tulane University
Department of Chemical & Biomolecular Engineering
New Orleans, LA
koc@tulane.edu

Online Resource 1: Supplementary methods, tables and figures

Supplementary methods

Flow cytometric analysis

Mesenchymal stem cells (MSCs) were expanded to passage 4 for flow cytometric analysis. Routine immunophenotyping was performed as we described in Madsen et al. (2020) with antibodies listed in Supplementary Table S1. Cells were labeled with phycoerythrin (PE)-conjugated monoclonal antibodies to detect human CD264 (clone #104918, R&D Systems, Minneapolis, MN, USA).

A Gallios analyzer equipped with Gallios software v1 (Beckman Coulter, Brea, CA, USA) was used to measure senescence-associated β-galactosidase (SA β-gal) activity in living cells with a flow cytometric assay adapted from Gong et al. (2009) and Noppe et al. (2009). Unless otherwise stated, all supplies were ordered from Thermo Fisher Scientific (Waltham, MA, USA). MSCs were trypsinized and resuspended in complete culture medium with antibiotics (CCMA) at a concentration of 1-3 x 10^6 cells/ml. The cells were incubated at 37 ºC in CCMA containing the pH modulator bafilomycin A1 (Sigma-Aldrich, St. Louis, MO, USA) at a concentration of 0.1 μM for 1 hour. Next, the β-gal substrate 9H-(1,3-dichloro-9,9-dimethylacridin-2-one-7-yl) β-D-galactopyranoside (DDAO galactoside) was added to the cells at a concentration of 0.02 mM, and the cells were incubated at 37 ºC for an additional hour. MSC samples were then washed twice with PBS and labeled with PE-conjugated CD264 monoclonal antibodies. Labeled cells were washed twice with PBS and resuspended in PBS at a concentration of 1-2 x 10^6 cells/ml for analysis. MSC samples were processed in tandem with unlabeled and isotype controls that were treated with bafilomycin A1. Spectral overlap was corrected by multicolor compensation.

Beckman Coulter Kaluza software v2.1 was used for *post hoc* flow cytometric analysis. The sample size for analysis was $n \geq 10{,}000$ cells. MSCs with fluorescence values greater than the 99% percentile of the fluorescence distribution for the isotype control were designated positive for antigen expression. Scatter properties and SA β-Gal activity of cell populations are reported as a ratio relative to the value for the parent culture.

Fluorescence-associated cell sorting (FACS)

MSCs at passage 4 were FACS sorted into CD264$^{-/+}$ cell populations based on our protocol described by Madsen et al. (2020). Specifically, the cells were exposed briefly to 0.25% trypsin/1 mM EDTA for 3 minutes. Trypsin was deactivated immediately with CCMA, and cells were resuspended in PBS at a concentration of 1-3 x 10^6 cells/ml for labeling. Following suspension in PBS, the MSCs were incubated for 30 minutes on ice and in the dark with monoclonal anti-CD264-PE antibodies (clone #104918, R&D Systems) at saturating conditions and 1 µl/ml ghost dye red 780 (Tonbo Biosciences, San Diego, CA, USA) to detect necrotic cells. Labeled cells were washed twice with PBS and then resuspended at 3 x 10^6 cells/ml in chilled 10% fetal bovine serum in phenol-free minimal essential media alpha with 100 units/ml penicillin and 100 µg/ml streptomycin for sorting.

The FACSMelody cell sorter equipped with FACSChorus software v3.0 (Becton Dickinson and Company, Franklin Lakes, NJ, USA) was employed for cell sorting and reanalysis. Labeled MSCs were processed in tandem with unlabeled, isotype-labeled and necrotic-positive controls. All sorted samples were ≥ 90% viable. The positive control for necrosis was obtained by incubating MSCs for 5 minutes at 90 °C. Multicolor compensation was performed with Becton Dickinson fluorescence control beads, and spectral overlap correction was validated with fluorescence-minus-one controls. Forward and side scatter gating was used to eliminate cellular debris and doublets, and ghost dye red 780 was used to gate viable cells. MSCs were sorted into CD264$^-$ and CD264$^+$ populations with the bottom and top 10-15% PE fluorescence, respectively. A small portion of sorted cells was reanalyzed to determine sort purity, mean forward scatter and mean side scatter. Sorted cells were collected in chilled CCMA for further experimentation.

Quantitative reverse transcription polymerase chain reaction (qPCR)

Single-stranded cDNA was generated from RNA using the High-Capacity cDNA Reverse Transcription Kit with RNase Inhibitor. TaqMan gene expression assays shown in Supplementary Table S2 were used with the TaqMan Fast Advanced Master Mix for qPCR. The selected assays have probes and/or amplicons that span exon junctions to not detect gDNA. A template-free control and an amplification-free control was used to check for the formation of primer dimers and to detect background fluorescence, respectively. Amplicon size was verified by running the samples on the Invitrogen E-Gel base using Invitrogen 4% agarose E-Gels with SYBR Safe and the Invitrogen E-Gel ultra-low range DNA ladder. Three reference genes for cellular aging were tested to normalize threshold cycle numbers: glyceraldehyde-3-phosphate dehydrogenase, glucuronidase beta and Pumilio RNA-binding family member 1 (*PUM1,* Gonzalez-Bermudez et al. 2019). *PUM1* was selected because it had the least amount of variation in expression between CD264$^-$ and CD264$^+$ samples.

Other assays

The differentiation potential of MSCs was determined 21 days after inducing osteo-, adipo- and chondrogenesis as we previously described (Russell et al. 2011). Alizarin Red S (Sigma-Aldrich) identified calcified extracellular matrix in osteogenic samples, AdipoRed (Lonza, Walkersville, MD, USA) stained lipid droplets in adipogenic samples, and Alcian Blue (Sigma-

Aldrich) detected matrix deposition of sulfated glycosaminoglycans in chondrogenic samples. To evaluate colony-forming efficiency, a small portion of the sorted cell populations were plated into T-flasks containing CCMA immediately after FACS and allowed to recover for 24 hours. The efficiency of MSCs to form colonies containing ≥ 50 cells was evaluated according to our protocol described in Barrilleaux et al. (2009), using crystal violet (Sigma-Aldrich) in methanol (Sigma-Aldrich) to visualize colonizes. The Senescence Cells Histochemical Staining Kit (Sigma-Aldrich) was used to assess SA β-gal activity in subconfluent MSCs at pH 6 in 12-well plates. Stained cells were imaged with a BioTek Cytation 5 Cell Imaging Multimode Reader (Agilent Technologies, Santa Clara, CA, USA) in bright field mode at 4X magnification. To estimate the percentage of SA β-gal^{+} (blue) cells, $n \geq 250$ cells per sample were counted in randomly selected areas of the wells. The positive control for β-gal activity was MSCs at pH 5 (Chen et al. 2007).

Other statistical analysis

Unless otherwise stated, a paired two-tailed Student's t test was used to assess differences between sorted CD264^{-} and CD264^{+} populations. Data are reported as mean ± standard error. Multiple linear regression was employed to test for significant differences in culture composition and gene expression. Regression analysis was performed with SAS Studio software v3.81 (copyright 2003, SAS Institute Inc., Cary, NC, USA). To compare gene expression estimated from RNAseq raw counts and qPCR threshold cycle numbers, the data were centered for pairwise comparisons and normalized to the same scale with Z-scores (Equations 1 - 6).

$$average\ \Delta C_t = average\ C_t\ for\ gene\ of\ interest - average\ C_t\ for\ housekeeping\ gene \quad (1)$$

$$relative\ gene\ expression = 2^{-(average\ \Delta C_t)} \quad (2)$$

$$centered\ count = count - mean\ count\ of\ the\ pair \quad (3)$$

$$sample\ mean = \sum(centered\ count\ of\ donor\ samples)/(number\ of\ donor\ samples) \quad (4)$$

$$sample\ standard\ deviation = \sqrt{\frac{\sum(centered\ count - sample\ mean)^2}{(number\ of\ donor\ samples - 1)}} \quad (5)$$

$$Z\text{-}score = \frac{centered\ count - sample\ mean}{sample\ standard\ deviation} \quad (6)$$

In Equations 3 - 6, "count" refers to the raw count from RNAseq or relative gene expression from qPCR. Sample calculations of Z-scores are provided in Supplementary Table S3. The vegan package (Dixon 2003) in R was used to perform a permutational multivariate analysis of variance in conjunction with a permutational multivariate analysis of dispersion (Anderson 2006) to test for differences in the centroid and dispersion of Z-score clusters.

The effectiveness of select genes to predict the CD264 classification of MSC samples was evaluated with confusion matrices constructed from the DESeq2 normalized counts of gene expression. For each gene of interest, the counts from donor samples were ranked from lowest to highest. CD264 classification of a sample was predicted as positive for upregulated genes during

cellular aging when the sample count was in the top 50% of the ranking and predicted as negative when the count was in the bottom 50%. The ranking was reversed for downregulated genes. A confusion matrix was constructed for each gene to compare actual and predicted CD264 classifications. There were four possible outcomes from the comparison: true positive, true negative, false positive and false negative. A sample was labeled as true positive when the actual and predicted classification was $CD264^+$; true negative, both classifications were $CD264^-$; false positive, predicted classification was positive for a $CD264^-$ sample; and false negative, predicted classification was negative for a $CD264^+$ sample. Positive and negative predictive values for each gene were calculated from the confusion matrix according to Equations 7 and 8.

$$Positive\ predictive\ value\ = \frac{true\ positives}{(true\ positives +\ false\ positives)} \tag{7}$$

$$Negative\ predictive\ value\ = \frac{true\ negatives}{(true\ negatives + false\ negatives)} \tag{8}$$

References

Anderson M J (2006) Distance-based tests for homogeneity of multivariate dispersions. Biometrics 62 (1):245-253. doi: 10.1111/j.1541-0420.2005.00440.x

Barrilleaux B L, Phinney D G, Fischer-Valuck B W, Russell K C, Wang G, Prockop D J, O'Connor K C (2009) Small-molecule antagonist of macrophage migration inhibitory factor enhances migratory response of mesenchymal stem cells to bronchial epithelial cells. Tissue Eng Part A 15 (9):2335-2346. doi: 10.1089/ten.tea.2008.0434

Chen J-H, Ozanne S E, Hales C N (2007) Methods of cellular senescence induction using oxidative stress. In: Tollefsbol T O (ed) Biological aging: methods and protocols. Humana Press, Totowa, NJ, pp 179-189.

Dixon P (2003) VEGAN, a package of R functions for community ecology. J Veg Sci 14 (6): 927-930. doi: 10.1111/j.1654-1103.2003.tb02228.x

Gong H, Zhang B, Little G, Kovar J, Chen H, Xie W et al (2009) β-Galactosidase activity assay using far-red-shifted fluorescent substrate DDAOG. Anal Biochem 386 (1):59-64. doi: 10.1016/j.ab.2008.11.031

Gonzalez-Bermudez L, Anglada T, Genesca A, Martin M, Terradas M (2019) Identification of reference genes for RT-qPCR data normalisation in aging studies. Sci Rep 9:13970. doi: 10.1038/s41598-019-50035-0

Madsen S D, Jones S H, Tucker H A, Giler M K, Muller D C, Discher C T et al (2020) Survival of aging CD264(+) and CD264(-) populations of human bone marrow mesenchymal stem cells is independent of colony-forming efficiency. Biotechnol Bioeng 117 (1):223-237. doi: 10.1002/bit.27195

Noppe G, Dekker P, de Koning-Treurniet C, Blom J, van Heemst D, Dirks R W et al (2009) Rapid flow cytometric method for measuring senescence associated β-galactosidase activity in human fibroblasts. Cytometry A 75 (11):910-916. doi: 10.1002/cyto.a.20796

Russell K C, Lacey M R, Gilliam J K, Tucker H A, Phinney D G, O'Connor K C (2011) Clonal analysis of the proliferation potential of human bone marrow mesenchymal stem cells as a function of potency. Biotechnol Bioeng 108 (11):2716-2726. doi: 10.1002/bit.23193

TABLE S1 MSC immunophenotype: antibodies and antigen expression.

Antibody[a]				MSC expression
**Antigen**	**Fluorochrome**	**Supplier**	**Clone number**	
CD11b	PC5	Beckman Coulter	Bear1	-
CD19	APC	Beckman Coulter	J3-119	-
CD79α	PC5	Beckman Coulter	HM47	-
CD34	PE	Beckman Coulter	581	-
CD45	FITC	Beckman Coulter	ALB12	-
HLA-II	FITC	BD Biosciences	G46-2.6	-
CD73	PC7	Invitrogen	TY/IL8	+
CD90	APC	BD Biosciences	SE10	+
CD105	PE	Invitrogen	SN6	+

[a]Fluorochrome-conjugated, anti-human monoclonal antibodies were used to immunolabel mesenchymal stem cells (MSCs).

TABLE S2 TaqMan gene expression assays for qPCR.

Gene	Assay ID[a]	Amplicon length (bp)
MAP1A	Hs00357973_m1	126
PTTG1	Hs00851754_u1	86
PUM1	Hs00472881_m1	77

[a]All assays are specific for *Homo sapiens* and have probes and/or amplicons that span an exon junction to avoid detection of gDNA.

TABLE S3 Sample calculations of Z-scores for *PTTG1* expression.

**RNAseq[a]**			**qPCR[a]**		
**Parameter[b]**	**Donor 5**		**Parameter[b]**	**Donor 10**	
	**CD264$^-$**	**CD264$^+$**		**CD264$^-$**	**CD264$^+$**
Raw count	5897	2705	$2^{-(\text{average } \Delta Ct)}$	6.45	2.01
Pair mean	4301		Pair mean	4.23	
Centered count	1596	-1596	Centered $2^{-(\text{average } \Delta Ct)}$	2.22	-2.22
Z-score[c]	0.94	-0.94	Z-score[c]	1.05	-1.05

[a]Representative data from donors 5 and 10 based on RNAseq raw counts and qPCR threshold cycle numbers (C_t).

[b]Parameters are calculated using Equations 1 – 6 in the Supplementary Material.

[c]To calculate the Z-score, the sample standard deviation is 1695 for donors 1 – 5 and 2.11 for donors 6 – 10. The sample mean is zero for both sets of donors.

TABLE S4 Representative quality assessment data for RNA preparation, sequencing and mapping.

**Category**	**Parameter**	**Range[a]**
RNA isolation	260nm/230nm	1.8-2.4
	260nm/280nm	1.8-2.4
	RIN	9.9-10
Library preparation	Avg cDNA size (bp)	320-360
Sequencing	Avg per sequence quality score	>30
	Avg per sequence GC content	49-53%
	Per base N content	0
Mapping	Percent mapped	94-96%

[a]*n* = 10 samples from 5 donors

A

RNAseq			qPCR		
Donor	Age	Sex	Donor	Age	Sex
1	29	male	6	24	male
2	30	female	7	24	female
3	30	female	8	26	male
4	36	female	9	27	female
5	37	male	10	28	male

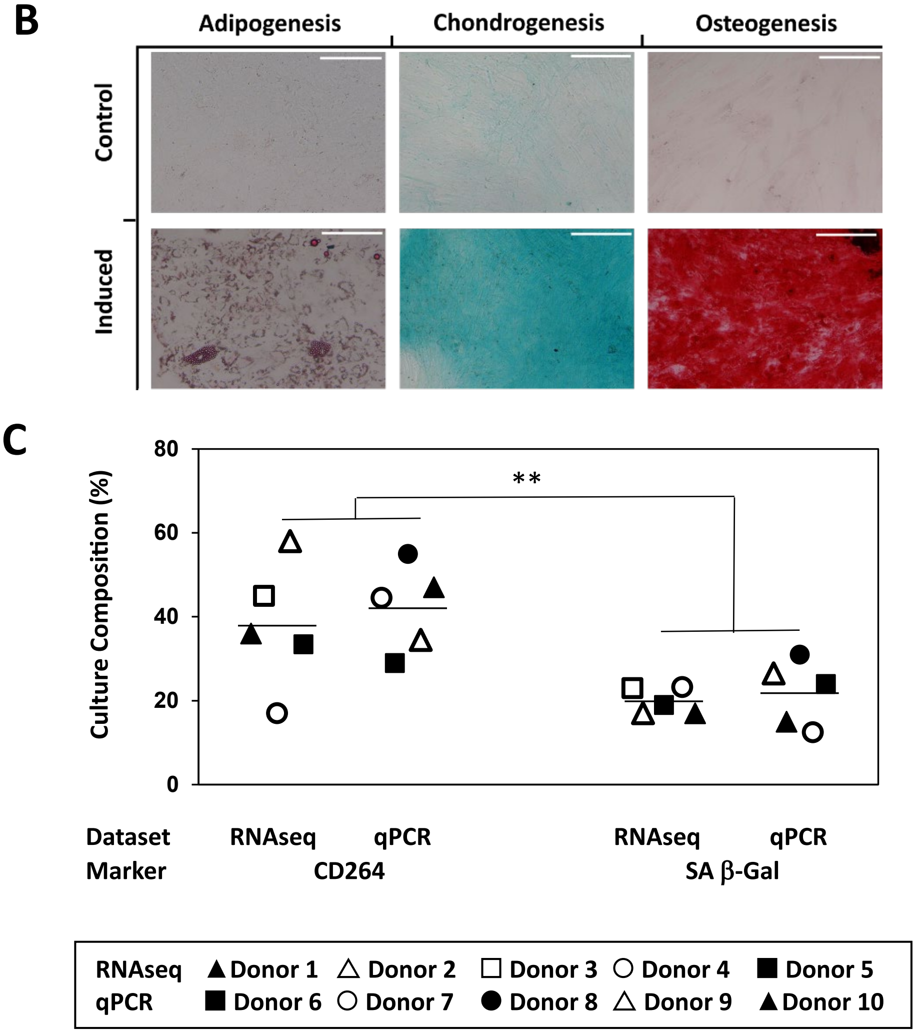

Fig. S1 Characterization of MSC parent cultures. **A** Donor demographics of MSCs employed in this study. **B** Trilineage differentiation of passage 4 MSCs cultured for 21 days in growth (control) or differentiation medium (induced). Images are of (1) monolayer cultures for adipo- and osteogenesis, and (2) outgrowth from a micromass culture for chondrogenesis. Cultures were stained with AdipoRed to detect lipids during adipogenesis; Alcian Blue, matrix deposition of sulfated glycosaminoglycans; and Alizarin Red S, mineralization during osteogenesis. Scale bar: 200 μm. **C** Percentage of cells in parent cultures expressing CD264 and staining positive for SA β-Gal in cultured-matched samples (n = 5 donors). Mean values depicted as bars. **p < 0.001 vs. SA β-Gal. Nomenclature: MSC, mesenchymal stem cell; qPCR, quantitative reverse transcription polymerase chain reaction; RNAseq, RNA sequencing; SA β-Gal, senescence-associated β-galactosidase

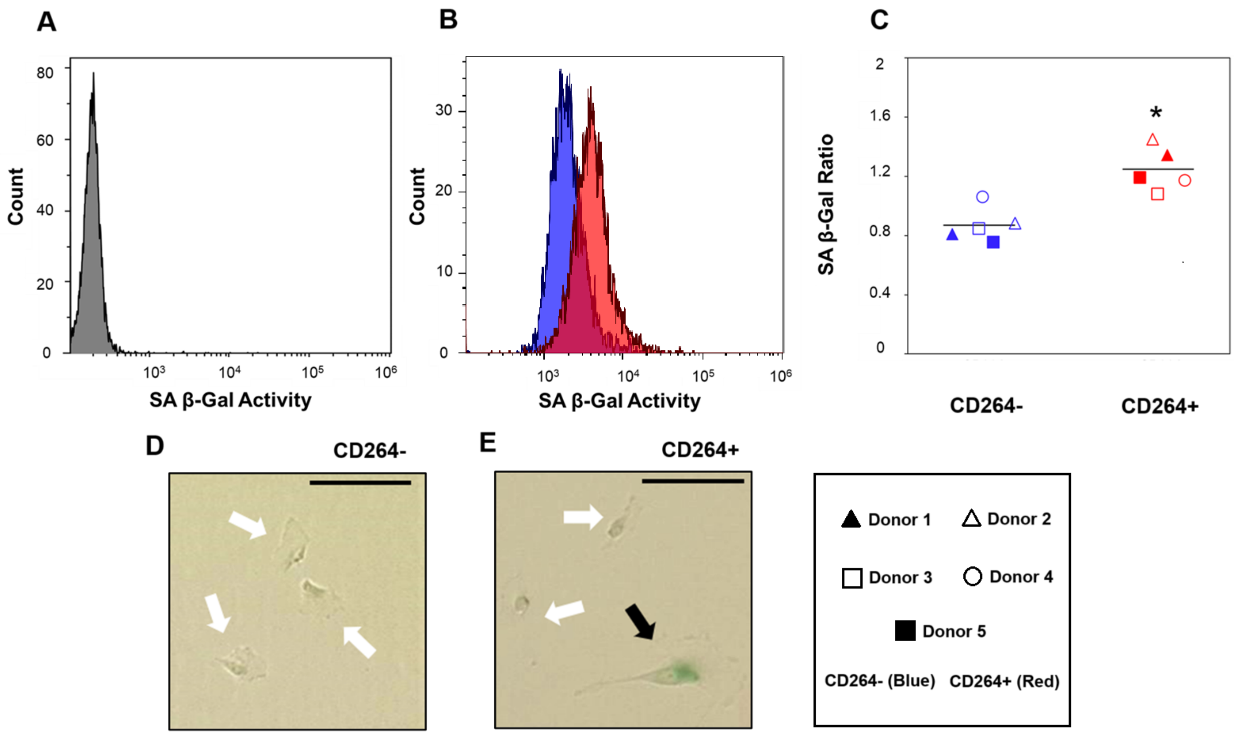

Fig. S2 SA β-Gal activity of CD264$^{-/+}$ MSCs. **A**-**C** Flow cytometric analysis of passage 4 MSCs exposed to the pH modulator bafilomycin A1 and the β-Gal substrate DDAO galactoside and then labeled with anti-CD264 monoclonal antibodies. The top and bottom 10% of CD264-expressing cells were selected for β-Gal analysis. **A**, **B** Representative histograms of SA β-Gal activity for (**A**) unstained control and (**B**) CD264^{-} (blue) and CD264^{+} (red) cells. **C** Mean fluorescence intensity ratio of CD264$^{-/+}$ SA β-Gal activity relative to parent culture. **D**, **E** Representative phase-contrast micrographs of FACS-sorted (**D**) CD264^{-} and (**E**) CD264^{+} MSCs after histochemical staining for SA β-Gal activity at pH 6. Arrows denote negative (white) and positive (black) β-Gal staining. Scale bars: 100 μm. *p < 0.05 vs. CD264^{-} MSCs. Nomenclature: DDAO galactoside, 9H-(1,3-dichloro-9,9-dimethylacridin-2-one-7-yl) β-D-galactopyranoside; FACS, fluorescence-activated cell sorting; MSC, mesenchymal stem cell; SA β-Gal, senescence-associated β-galactosidase

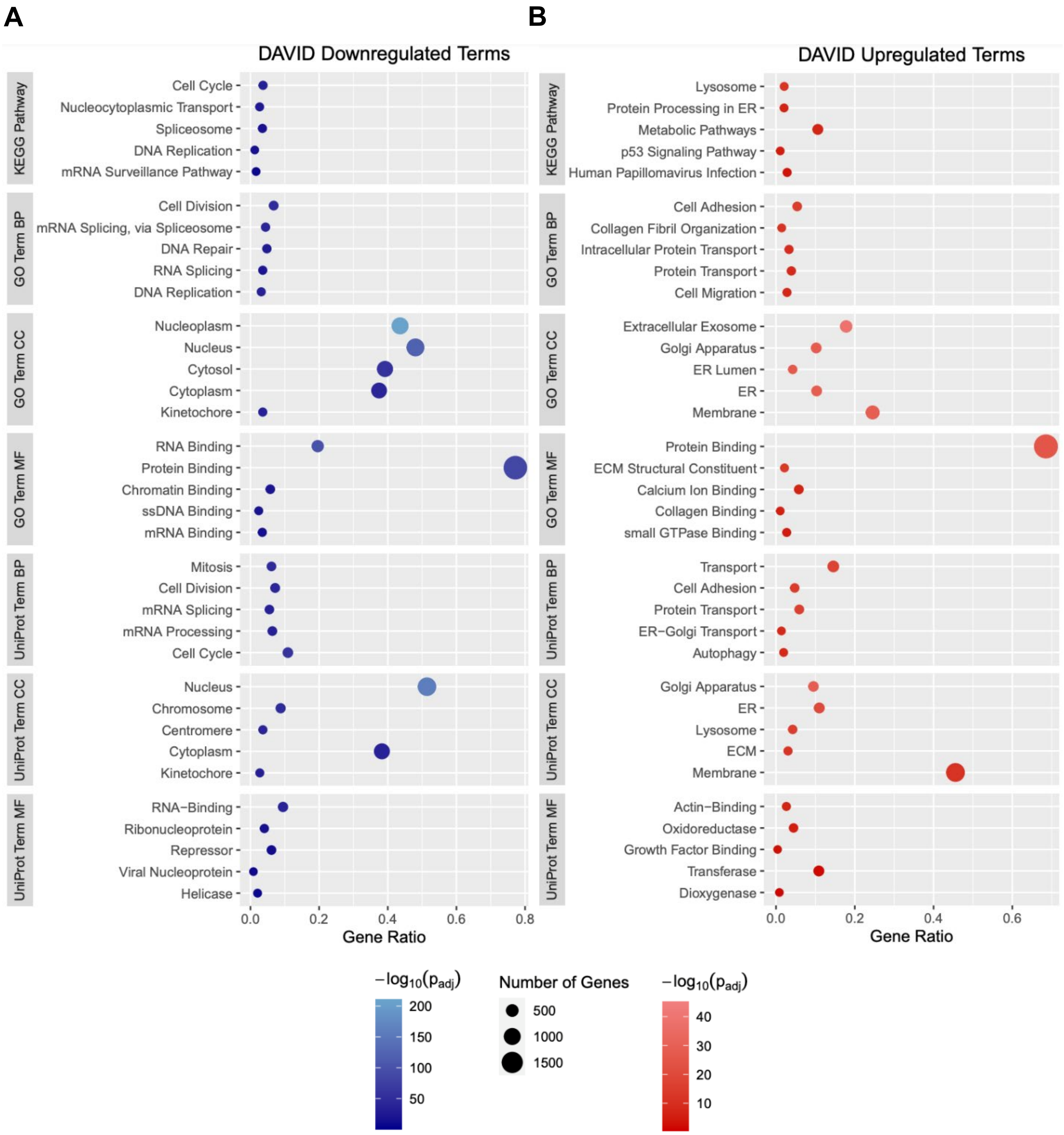

Fig. S3 DAVID analysis of DEGs in CD264$^{-/+}$ MSCs. Enriched KEGG pathways, GO terms and UniProt terms were determined for all DEGs with BH $p_{adj} < 0.1$ for CD264$^{-/+}$ MSCs at passage P4. **A**, **B** Dot plots of the most significantly downregulated (**A**, blue) and upregulated (**B**, red) terms in CD264^{+} MSCs. Nomenclature: BP, biological process; CC, cellular component; DAVID, Database for Annotation, Visualization and Integrated Discovery; ECM, extracellular matrix; ER, endoplasmic reticulum; GO, Gene Ontology; KEGG, Kyoto Encyclopedia of Genes and Genomes; MF, molecular function; MSC, mesenchymal stem cell; ss, single stranded; UniProt, Universal Protein Resource

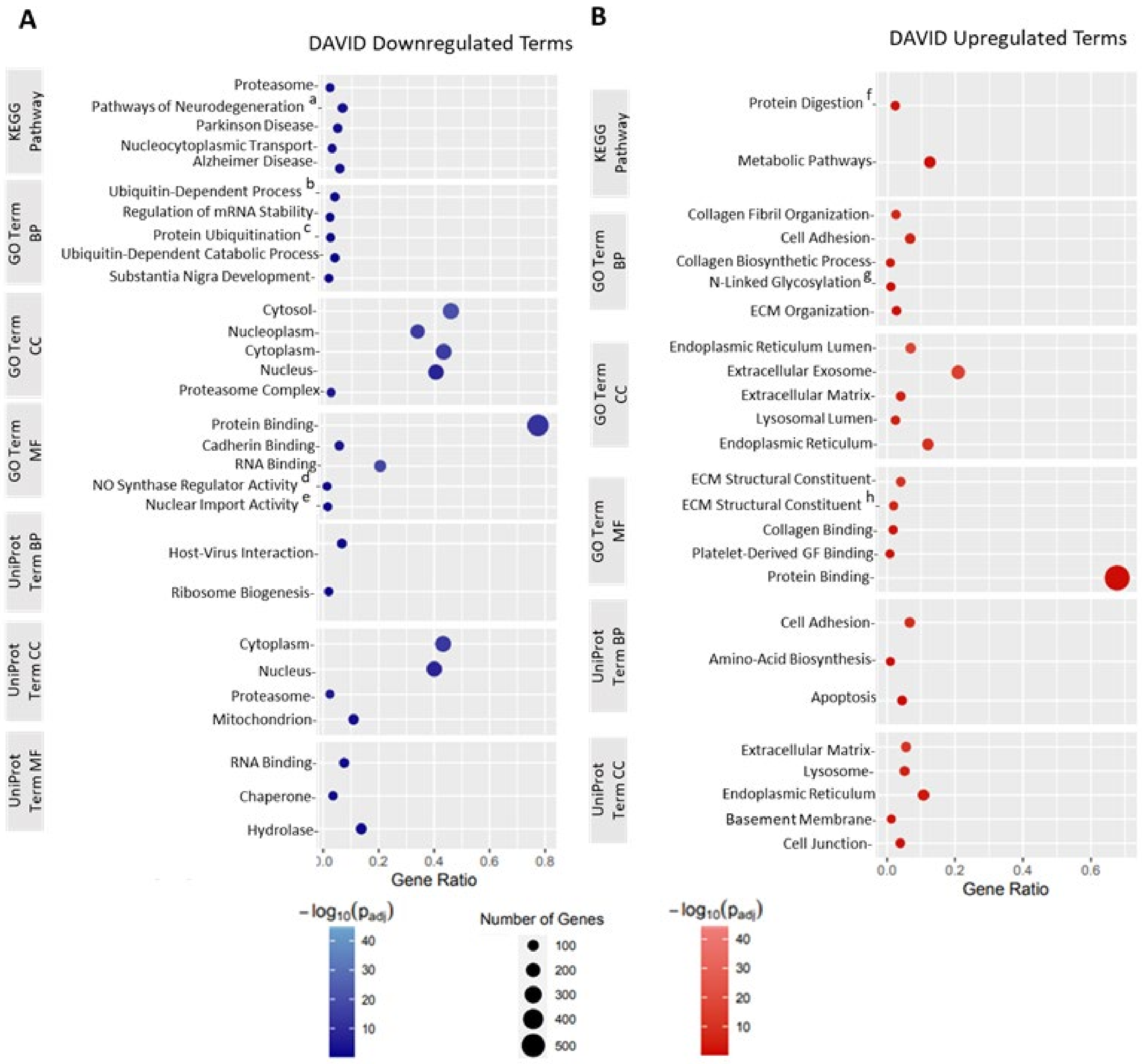

Fig. S4 DAVID analysis of unique DEGs in $CD264^{-/+}$ MSCs unreported in previous RNAseq studies of early- vs. late-passage MSCs. Enriched KEGG pathways, GO terms and UniProt terms were determined for nonoverlapping DEGs with BH $p_{adj} < 0.1$ for passage 4 $CD264^{-/+}$ MSCs show in Figures 3A and B. **A**, **B** Dot plots of the most significantly downregulated (**A**, blue) and upregulated (**B**, red) terms for $CD264^{+}$ MSCs. There were no significantly unregulated UniProt terms for molecular function. [a]Pathways of neurodegeneration - multiple diseases, [b]proteasome-mediated ubiquitin-dependent protein catabolic process, [c]positive regulation of protein ubiquitination, [d]nitric oxide synthase regulator activity, [e]nuclear import signal receptor activity, [f]protein digestion and absorption, [g]protein N-linked glycosylation via asparagine, [h]extracellular matrix structural constituent conferring tensile strength. Nomenclature: GF, growth factor. See caption for Figure S3 for other nomenclature

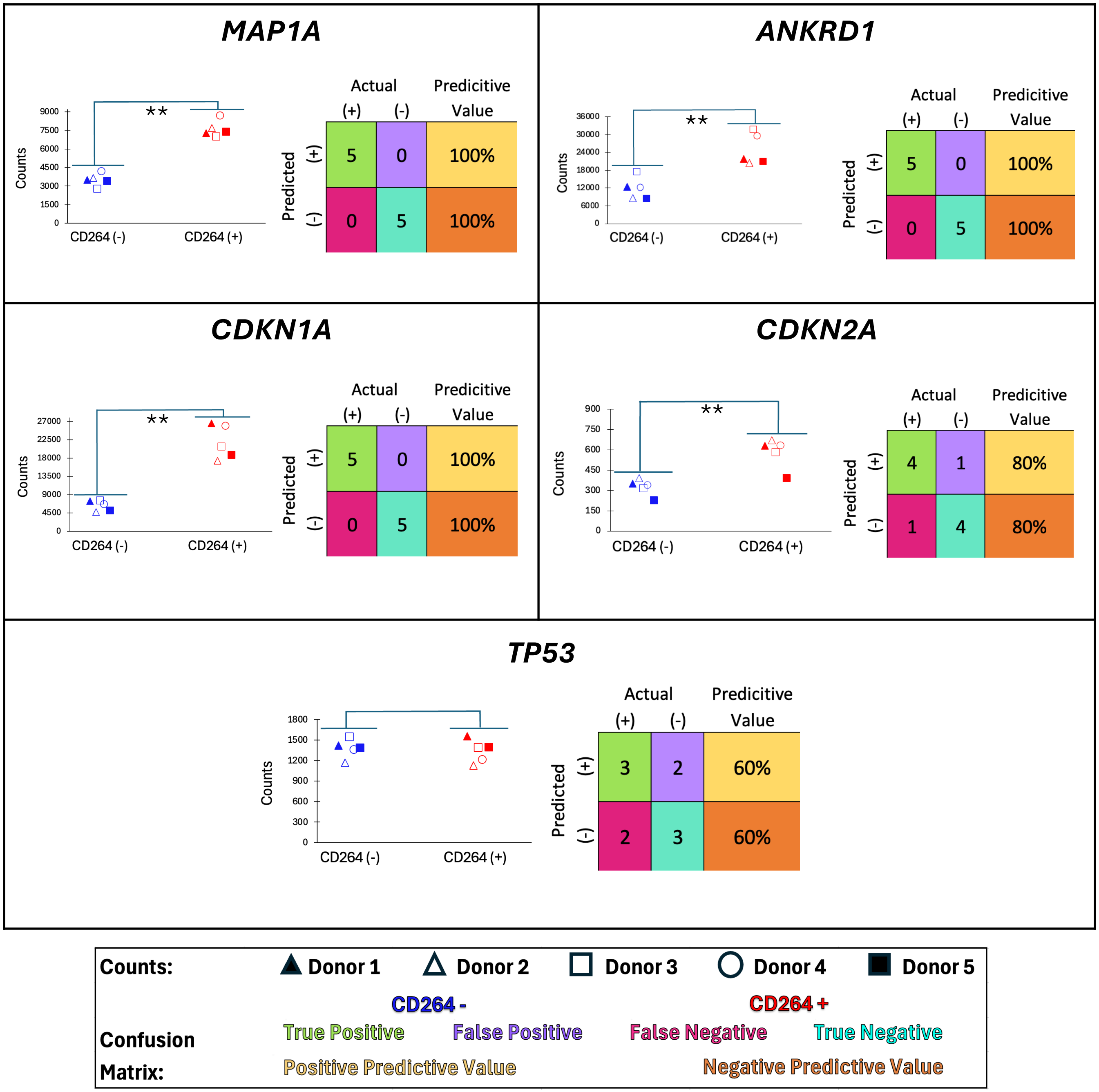

Fig. S5 Predicting CD264 classification of MSCs with gene expression of *MAP1A*, *ANKRD1*, *CDKN1A*, *CDKN2A* and *TP53*. Positive and negative predictive values for each gene of interest were evaluated with a confusion matrix constructed from DESeq2 normalized counts as described in the Supplementary Methods. The confusion matrix compared the actual CD264 classification of MSC samples with the predicted classification based on gene expression (n = 10 RNA sequencing samples). Positive predictive value = true positives/(true positives + false positives). Negative predictive value = true negatives/(true negatives + false negatives). Nomenclature: *ANKRD1*, ankyrin repeat domain 1; *CDKN1A*, cyclin-dependent kinase inhibitor 1A; *CDKN2A*, cyclin-dependent kinase inhibitor 2A; *MAP1A*, microtubule-associated protein 1A; and *TP53*, tumor protein p53. ** Bonferroni $p_{adj} < 0.05$ vs. $CD264^{-}$ MSCs. See Table 1 for specific p_{adj} values.

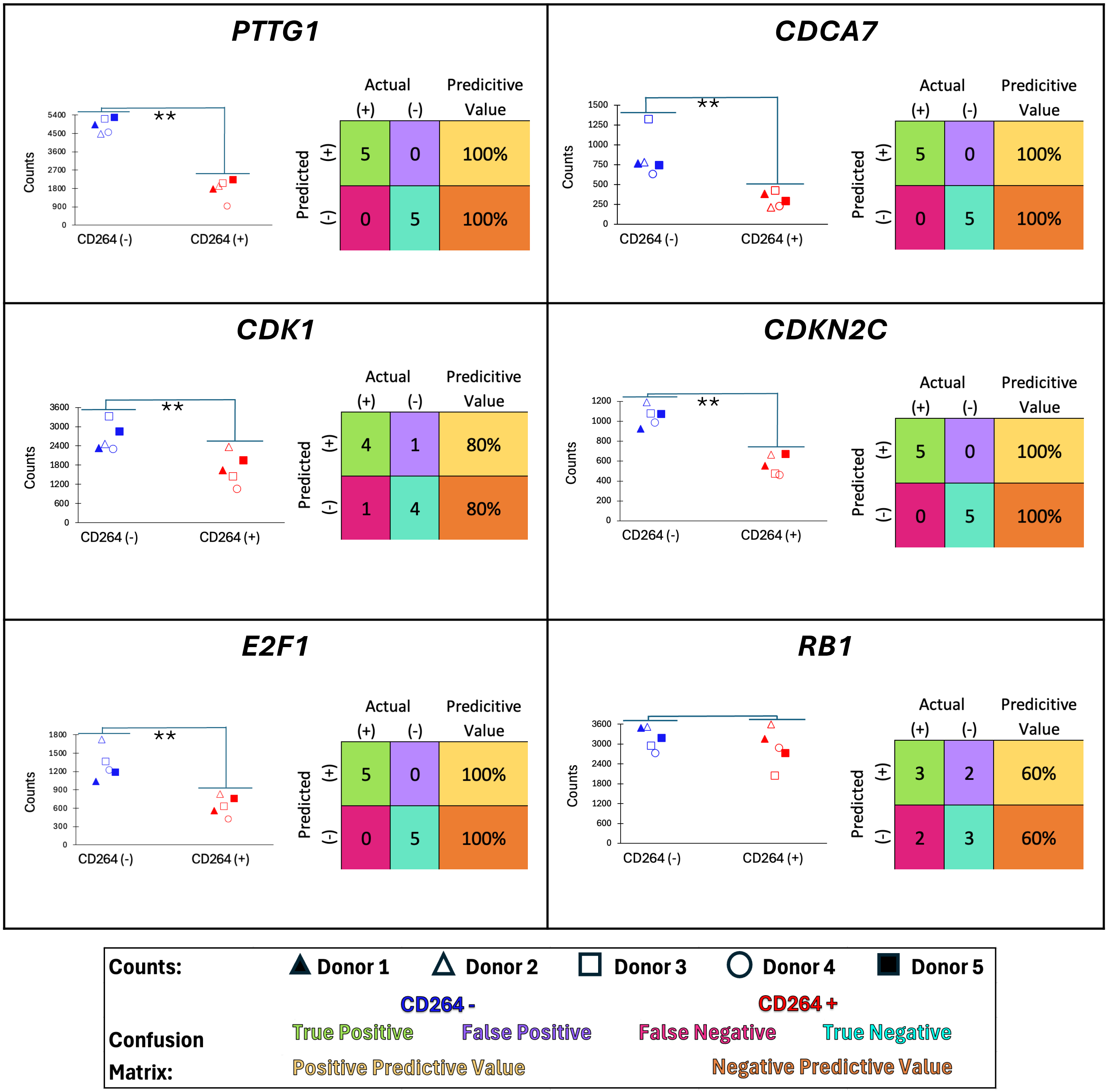

Fig. S6 Predicting CD264 classification of MSCs with gene expression of *PTTG1*, *CDCA7*, *CDK1*, *CDKN2C*, *E2F1* and *RB1*. Positive and negative predictive values for each gene of interest were evaluated with a confusion matrix constructed from DESeq2 normalized counts as described in the caption for Figure S5 (*n* = 10 RNA sequencing samples). Nomenclature: *PTTG1*, pituitary tumor-transforming gene 1; *CDCA7*, cell division cycle-associated protein 7; *CDK1*, cyclin-dependent kinase 1; *CDKN2C*, cyclin-dependent kinase inhibitor 2C; *E2F1*, E2F transcription factor 1; and *RB1*, retinoblastoma 1. ** Bonferroni $p_{adj} < 0.05$ vs. CD264$^-$ MSCs. See Table 1 for specific p_{adj} values.
